# Body Language Influences on Facial Identification at Passport Control: An Exploration in Virtual Reality

**DOI:** 10.1177/2041669520958033

**Published:** 2020-10-18

**Authors:** Hannah M. Tummon, John Allen, Markus Bindemann

**Affiliations:** School of Psychology, University of Kent, Canterbury, United Kingdom

**Keywords:** face matching, identification, nonverbal behaviour, body language, passport control, virtual reality

## Abstract

Person identification at airports requires the matching of a passport photograph to its bearer. One aim of this process is to find identity impostors, who use valid identity documents of similar-looking people to avoid detection. In psychology, this process has been studied extensively with static pairs of face photographs that require identity match (same person shown) versus mismatch (two different people) decisions. However, this approach provides a limited proxy for studying how other factors, such as nonverbal behaviour, affect this task. The current study investigated the influence of body language on facial identity matching within a virtual reality airport environment, by manipulating activity levels of person avatars queueing at passport control. In a series of six experiments, detection of identity mismatches was unaffected when observers were not instructed to utilise body language. By contrast, under explicit instruction to look out for unusual body language, these cues enhanced detection of mismatches but also increased false classification of matches. This effect was driven by increased activity levels rather than body language that simply differed from the behaviour of the majority of passengers. The implications and limitations of these findings are discussed.

International airports provide key entry points for people into other countries, with heightened security measures in recent years leading to greater interaction between passengers and security personnel (Trainer, 2017). Admission of entry relies critically on the routine identification of a large volume of passengers. This is typically achieved by identification from photographic documentation, by comparing the article image with its bearer. A key security issue here concerns the detection of identity impostors, who travel on legitimate documents of someone who is similar in facial appearance to avoid detection at passport control (Stevens, in press). This identification process has been studied widely in Psychology through unfamiliar face matching. In psychological experiments, pairs of facial images of unknown people are compared to be classified either as an identity match (i.e., as depicting the same person) or an identity mismatch (i.e., two different people). Extensive laboratory research has highlighted the difficulty of this task, across a range of conditions (for reviews, see Fysh & Bindemann, 2017a; Jenkins & Burton, 2008, 2011; Robertson et al., 2015, 2019). This difficulty persists even for trained and experienced passport control personnel (White et al., 2014; Wirth & Carbon, 2017).

An important gap exists, however, between these laboratory experiments on face matching and the real world of person identification at airports. Almost all existing research has investigated face matching by presenting pairs of cropped faces on plain backgrounds. This approach has been successful for advancing understanding of how a range of factors affect face matching, such as variation in a person’s appearance (Bindemann & Sandford, 2011; Megreya et al., 2013; Ritchie & Burton, 2017), the addition of disguise (Henderson et al., 2001; Kramer & Ritchie, 2016; Wirth & Carbon, 2017), and individual differences in the ability of observers (e.g., Bindemann et al., 2012; Bobak, Dowsett, et al., 2016; Bobak, Hancock, et al., 2016; Megreya & Burton, 2006). From this body of research, it is easy to understand why this approach has become prevalent in the study of face matching, because it allows for tight control of experimental variables.

However, this simplistic approach offers a limited proxy for understanding how factors that present in more complex settings interact with this task (Ramon et al., 2019). One method for investigating face matching during such real-world social interaction is to conduct field experiments. Only a few studies have examined face matching under these kinds of circumstances, for example, by examining the ability of supermarket cashiers (Kemp et al., 1997) or passport officers (White et al., 2014) to identify people from photo-ID during face-to-face interaction, or to examine the benefit of viewing multiple images of a person for face identification (Ritchie et al., 2020). However, such experiments are logistically challenging, and participants’ behaviours are difficult to control systematically. Consequently, additional measures, such as double-blind procedures, are taken to *prevent* intrusion of potentially interesting variables that might present in real-world experiments. Equally, face matching is difficult to study systematically in occupational field settings, such as at passport control, owing to the security-sensitive nature of this task.

In this study, we apply a new methodology in an attempt to overcome these limitations, by investigating face identity matching with virtual reality (VR). This technology enables the simulation of complex and detailed environments that can be strictly controlled for the purpose of experiments. This novel approach therefore allows for the study of factors that may impact real-world person identification, but that conventional laboratory experiments cannot easily address. We have recently developed and validated this VR approach through a series of experiments. These demonstrate that VR avatars can preserve identity information from real faces and that matching of pairs of such avatar faces also reflects similar cognitive processes to the matching of photographs of real faces (Tummon et al., 2019). This approach is now beginning to be used in the study of face perception with some promising results. For example, a recent study demonstrates that dynamic visual exploration of person avatars in VR facilitates face learning, resulting in more robust recognition (Bülthoff et al., 2019). Here, we employ this approach to provide insight into a factor that controlled laboratory studies have not investigated in this domain, by examining whether nonverbal behaviour in the form of variation in body movement affects decision-making in a security-critical face-matching task.

The psychological literature demonstrates that body language can have substantial impact on interpersonal interaction and judgements (e.g., Burgoon et al., 2011; Knapp et al., 2013). In passport control settings, a heuristic technique utilising body language may be pivotal, for example, by seeking out those who appear to be behaving unusually. Consequently, substantial effort has been invested in programmes that train staff to look for such nonverbal cues in aviation settings, such as the Screening of Passengers by Observation Techniques programme in the United States (see United States Government Accountability Office, 2010). The aim of these programmes has been to equip personnel to identify persons who are seeking to evade detection or pose potential threats, by looking for signs of suspicious and unusual behaviour. It is not clear whether this has enhanced security (United States Government Accountability Office, 2013), but personnel training to spot suspicious or unusual behaviour of passengers at airports continues to be sought by aviation authorities (e.g., Majumder, 2019) and is advertised as an important skill for aspiring passport officers (e.g., Guy, 2019a; TARGETjobs, 2019). For these officers, the day-to-day checking of passports also involves the observing and questioning of passengers, and looking for suspicious behavioural indicators can be a particularly engaging aspect of the role (e.g., Guy, 2019b). To date, however, there are few studies that have systematically examined the impact of unusual body language on the face identifications that are required at passport control.

More broadly, there is already some evidence that information from the body affects the identity matching of unfamiliar people. For example, although the face outperforms the body in identity matching tasks when these types of stimuli are presented in isolation, accuracy is best when both sources of information are available (Rice, Phillips, & O’Toole, 2013). This effect appears to be amplified by increasing viewing distance, which shifts observers’ reliance on identity information further towards the body (Hahn et al., 2016). The utility of combining facial and body information has also been highlighted in identity sorting tasks, where people are easier to distinguish when the whole person is shown than from faces and bodies shown in isolation (Balas & Pearson, 2017). Remarkably, however, observers’ self-reports of usage are much lower for body features than internal facial features when making identifications. This is an important finding as it suggests that observers often remain unaware of their reliance on body information as identity cues when facial information is insufficient (Rice, Phillips, Natu, et al., 2013). Further evidence for the integration of the body with facial information in person identification comes from paradigms that present people in motion. This research shows that facial information is prioritised over body cues when static stimuli are observed, but both are utilised in a more balanced manner when dynamic stimuli are used, resulting in superior person identification accuracy (O’Toole et al., 2010; Simhi & Yovel, 2016; Yovel & O’Toole, 2016).

These findings highlight the role of the body in person identification but do not address how specific body *language*, that is not indicative of identity but may reflect a hidden motivation, might affect identification in security settings. Impostors seeking to avoid detection at airports might, for example, betray their intention through behavioural cues of anxiety, such as restless fidgeting, body posture changes, and limb movements (e.g., DeTurck & Miller, 1985; Ekman & Friesen, 1969). In turn, observers often assume that increases in the frequency and intensity of body language behaviours, such as postural shifts and movement, are indicators of deception (Akehurst et al., 1996; Bogaard et al., 2016; Hartwig & Granhag, 2015; Strömwall & Granhag, 2003). Crucially, this is found not only with laypersons, such as members of the general public, but also police (Akehurst et al., 1996; Bogaard et al., 2016; Masip & Herrero, 2015; Vrij et al., 2006), customs (Kraut & Poe, 1980; Vrij & Semin, 1996), and immigration officers (Granhag et al., 2005; see Strömwall et al., 2004, for a review).

In the context of face matching at passport control, where ID checking is carried out by such professionals, the conundrum that such nonverbal cues pose becomes apparent from consideration of how these might influence identification decisions. On one hand, exhibition of unusual body language in queueing travellers could lead the ID checker to enhance their scrutiny during the identification check. This might increase accuracy generally on trials in which unusual body language is displayed, leading to increased accuracy for both identity mismatches and matches in this condition. This line of reasoning receives support from studies showing that face-matching accuracy improves as more time for this task becomes available (e.g., Bindemann et al., 2016; Fysh & Bindemann, 2017b; Özbek & Bindemann, 2011; White et al., 2015; Wirth & Carbon, 2017).

Alternatively, it is possible that unusual body language that differs from the majority of passengers might be seen as a specific indicator that an identity impostor is present. While the detection of these mismatching face pairings is a specific concern for person identification at passport control, these cases also occur with less frequency than identity matches (Bindemann et al., 2010; Fysh & Bindemann, 2017b, 2018a; Papesh & Goldinger, 2014; Susa et al., 2018). In the experiments reported here, mismatch frequency was therefore also kept low. Under these conditions, unusual nonverbal behaviour such as body language that differs from the majority of passengers might be seen as an additional indicator for mismatch face pairs by virtue of their shared infrequency characteristic. Consequently, if observers are sensitive to unusual body language, then this could also lead specifically to the enhanced detection of identity mismatches, whereas the classification of matches might be unaffected.

On the other hand, it is also possible that, instead of improving accuracy per se, unusual body language biases the classification of face pairs. If these body language cues are adopted as a simple heuristic to identify impostors, thereby reducing the scrutiny of facial information in these identity mismatches, then identity matches that exhibit such body language might be misclassified as mismatches, too. In this case, unusual body language would increase the proportion of both mismatches *and* matches that are classified as depicting two different people. Such biases are evident when other real-world variables are introduced into face-matching tasks. Presenting faces in the context of photo-identity documents, for example, biases observers towards making match decisions, thereby reducing detection of mismatches (Feng & Burton, 2019; McCaffery & Burton 2016). And face-matching decisions can be biased either towards match or mismatch responses by simple onscreen labels that suggest which type of face pair might be shown, mimicking information provided by Automated Border Control systems, even when observers are instructed to ignore this information (Fysh & Bindemann, 2018a).

In the experiments reported here, we employ VR to investigate these possibilities. For this purpose, participants were immersed in a virtual airport environment as passport control officers, who were required to make identification decisions for a queue of passengers in an arrivals hall. These passengers were equipped with an idle mode that creates small shifts in body posture when a person is stationary, to increase observers’ sense of realism in VR. In a proportion of these passengers, the idle level was raised to simulate more restless body language. The question of main interest here was whether this alternate display of body language would be perceived as unusual in this context and would affect face-matching decisions.

As a side aim, we sought to investigate whether these effects depend on explicit instruction to attend to body language during person identification. Considering that observers can remain unaware of utilising the body during person identification (Rice, Phillips, Natu, et al., 2013), and that body language can influence observers without their awareness more widely (e.g., Engelen et al., 2018; Hall et al., 2019; Mandal, 2014; Tamietto et al., 2009), it is possible that differences in body language influence face matching when no specific instructions are provided to attend to this information. This is investigated in Experiments 1 and 2. Subsequent experiments examine face matching when explicit instructions to monitor body language are given.

## Experiment 1

This experiment investigated whether unusual body language influences person identification from the face in a matching task. At real-life passport control, officers would be positioned in front of queueing passengers, to compare their faces to their passport photographs. The VR airport of this study replicated this setup, with participants standing within a booth looking towards a queue of person avatars. To mimic real life, participants compared each of these three-dimensional (3D) avatars to a respective two-dimensional (2D) face portrait, which was displayed on a passport-style ID card, to determine whether this presented an identity match or mismatch to its bearer. The avatars were equipped with body language that someone might exhibit naturally while waiting to be processed at passport control. Thus, they were programmed to look around and shift in their stance occasionally. For most avatars, this animation was performed at idle; speed, which represented a normal level of animation in this study. In a subset of avatars, however, these activity levels were increased to represent restless and lively waiting behaviours. We then sought to determine how these increases in body language affected classification of identity matches and mismatches.

One possible outcome is that unusual body language in queuing travellers alerts the ID checker to enhance their scrutiny during the identity check. This could generally enhance classification of identity matches and mismatches with unusual body language. However, considering that both unusual body language and identity mismatches occur infrequently, the combination of these factors might also lead to an enhanced detection of identity mismatches only, by highlighting these specific cases. Finally, instead of improving accuracy per se, it is also possible that unusual body language biases the classification of face pairs, by increasing the proportion of both mismatches and matches that are classified as depicting two different people when unusual body language is displayed by an avatar.

### Method

#### Participants

The participants consisted of 30 Caucasian students from the University of Kent (5 male, 25 female), with a mean age of 20.5 years (*SD* = 5.0 years). All participants reported normal or corrected-to-normal vision and completed the experiment in exchange for course credit. As with all experiments in this study, owing to the use of VR equipment, no persons with epilepsy or who were liable to motion sickness were recruited. Before immersion in the VR environment, participants were briefed about potential side effects of using VR, such as discomfort from wearing the headset and symptoms of motion sickness, and health and safety procedures.

#### Stimuli

**Figure 1.**
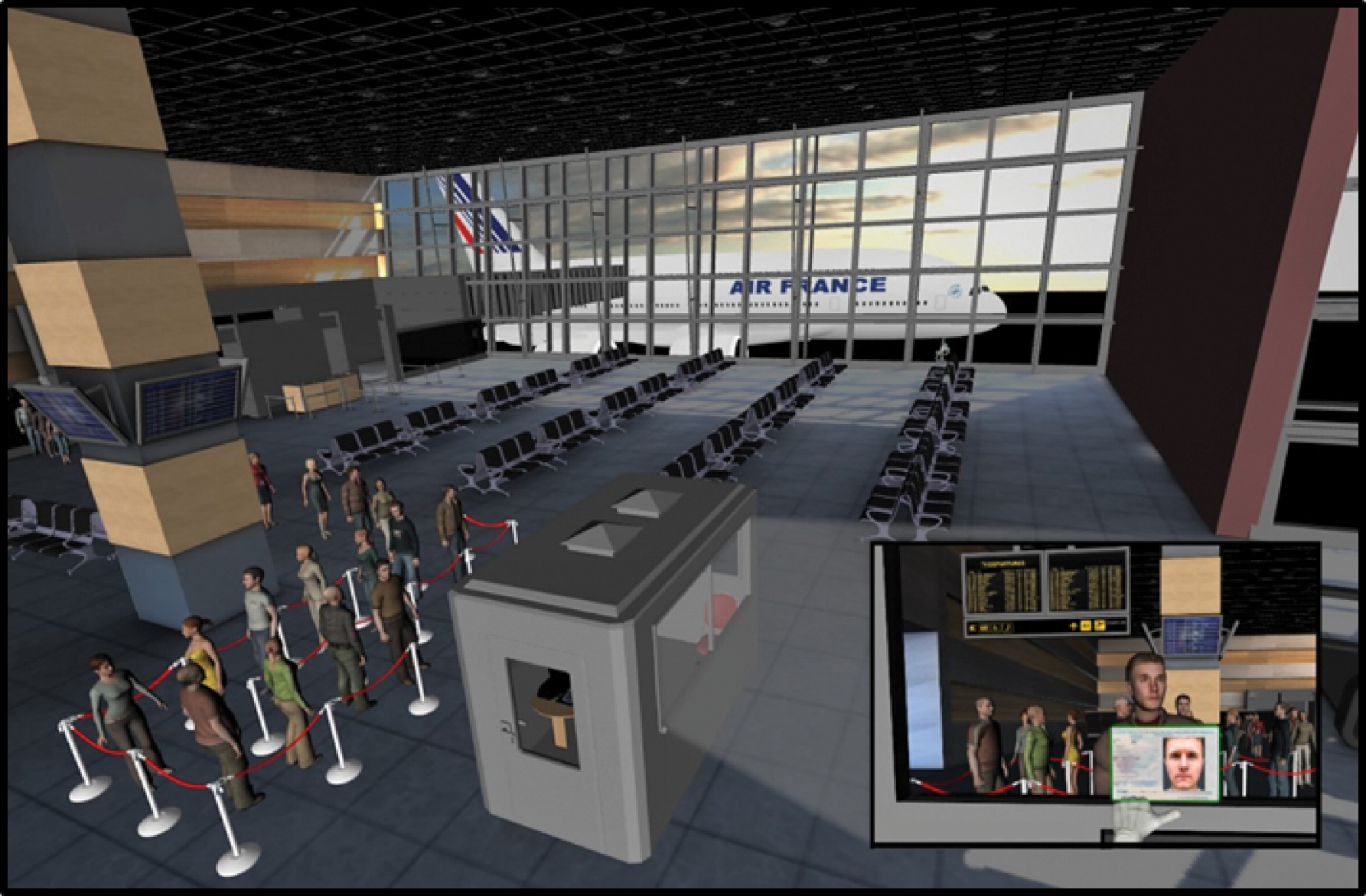
The virtual reality environment. The inset demonstrates the forward-facing view from inside the passport control booth.

The passport control environment was constructed by positioning 3D objects within a prebuilt 3D airport hall model (https://www.turbosquid.com/3d-models/airport-departures-lounge-3d-model/626226). This model was built in 3ds Max and used VRay for rendering. The completed passport control environment consisted of a booth area in which the participants were standing, equipped with a desk, chair, and computer. These objects were added to improve the realism of the booth, and so response button instructions could be overlaid on a virtual computer screen inside the passport control booth. This booth was situated inside the airport hall with other visual cues, such as departure boards and a waiting aeroplane, which were clearly visible to participants. The environment is illustrated in Figure 1.

The person stimuli consisted of 200 animated 3D avatars of 100 identities (2 avatars per identity). The 3D avatars were created by combining 2D photographs of real faces, which depicted professional German sportspeople of Caucasian ethnicity and an approximate age of between 20 and 35 years, with an avatar body from an existing database (see Tummon et al., 2019). Using graphics software (Artweaver 5), the internal features of a face photograph were mapped onto the features of the avatar’s face area, with the edges smoothed and skin colour adjusted to blend the graphics. A wide range of base bodies were selected when creating the avatars, with equal numbers of male and female identities. In addition, clothing was different across all avatars to ensure that the stimulus set suitably varied in body appearance.

To create identity match and mismatch pairs, a 2D face portrait was captured from one avatar of each identity to create a passport image, sized to 438 × 563 pixels at a resolution of 150 ppi, and embedded on a passport-style card. For matches, this passport-style card was paired with the second avatar of an identity for the experiment. For identity mismatches, an avatar was paired with a 2D face portrait of a similar-looking identity, matched for gender and approximate age. To provide a closer proximate to real-world conditions, mismatches occurred with much lower frequency than matches (e.g., Bindemann et al., 2010; Fysh & Bindemann, 2017b, 2018a; Papesh & Goldinger, 2014; Susa et al., 2018). Thus, 94 matches and 6 mismatches were created in this manner for the experiment. The 94 match trials were broken down further in 88 noncritical trials and 6 critical match trials, which were used as a direct comparison for the mismatches. The avatar pairings for the critical matches were selected to be comparable to the mismatches for accuracy (mean accuracy matches = 68.1%, *SD* = 21.7; mean accuracy mismatches = 65.7%, *SD* = 22.0), based on data from a previous study, *t*(119) = 0.77, *p* = .44, *d* = 0.11 (Tummon et al., 2019). Overall, this process resulted in 100 unique stimulus pairings. Avatars were not repeated across stimulus pairings.

The avatars were equipped with a built-in animation ( idle1 in Vizard) producing shifts in their stance while they were queuing in the airport. To manipulate body language, the scale factor of this animation was adjusted so that selected avatars were moving with different levels of activity (i.e., completing the animation cycle in differing durations). Three activity levels were used, which corresponded to an animation scale factor of 1 for the idle condition, in which a cycle lasted 13.3 seconds before being repeated, and of 2 (6.7 seconds per cycle) and 3 (4.4 seconds per cycle) for the restless and lively conditions, respectively. In all of the 88 noncritical trials, the avatars displayed idle body language. In the remaining cases, two of the critical matches and mismatches displayed idle, lively or restless body language. In the experiment, these activity manipulations were rotated around the critical match and mismatch trials across participants for a counterbalanced design.

#### Procedure

The experiment was controlled using Vizard 5 software. During the experiment, participants were immersed in a VR passport control environment with an HTC Vive headset with a resolution of 1,080 × 1,200 pixels per eye. Two handheld controllers enabled participants to interact with the environment and respond to the stimuli. In the VR airport environment, the 3D avatars approached from the back of an airport hall and proceeded to queue around rope barriers. One at a time, they walked towards the passport control booth, where the participants were positioned inside the VR environment, and waited to be processed. The corresponding 2D face portrait for each avatar passenger appeared on a passport-style photo card, which could be picked up and moved with the VR controllers. This enabled participants to hold the passport in any position necessary to facilitate an identity comparison, for example, close to the face of the animated avatar (see inset of Figure 1). Participants pressed the thumb pad of the right controller to report an identity match or the thumb pad of the left controller to report an identity mismatch. Once a response was given, the avatar walked away and the photo on the card changed to the one corresponding for the next avatar in line as it approached the desk. Participants continued making these match or mismatch decisions until the whole queue had been processed.

While queuing and standing at the desk, the avatars shifted in their stance through the avatars’ built-in animation, which was initiated at a random starting point in the cycle to prevent synchronised motion. Once at the desk area, the scale factor of this animation was adjusted so that selected avatars were moving with different levels of activity, corresponding to idle, restless or lively body language. The first 10 trials of the queue always consisted of 10 noncritical match trials to accustom participants to the idle activity level. The critical trials were then randomly distributed throughout the last 90 trials. Participants were informed at the beginning of the experiment that mismatch frequency would be low but were not made aware of variation in body language. They were not given any time restrictions in which to complete the task to encourage accurate performance.

Following the VR task, participants completed a questionnaire to report any differences in animation that they might have noticed. The purpose of the questionnaire was to examine participants’ awareness of the activity-level manipulation (i.e., whether they correctly perceived three levels of activity). Participants were first asked if they noticed anything unusual during the experiment, providing opportunity for them to report freely without being led to suspect differences in body language. Secondly, they were informed that some avatars may have been moving at different speeds (activity levels) and were asked to report how many they had perceived throughout the experiment. Finally, they reported the relative speeds of the perceived number of activity levels using sliding scales to verify the response given in the previous question.

### Results

#### Percentage Accuracy

**Figure 2.**
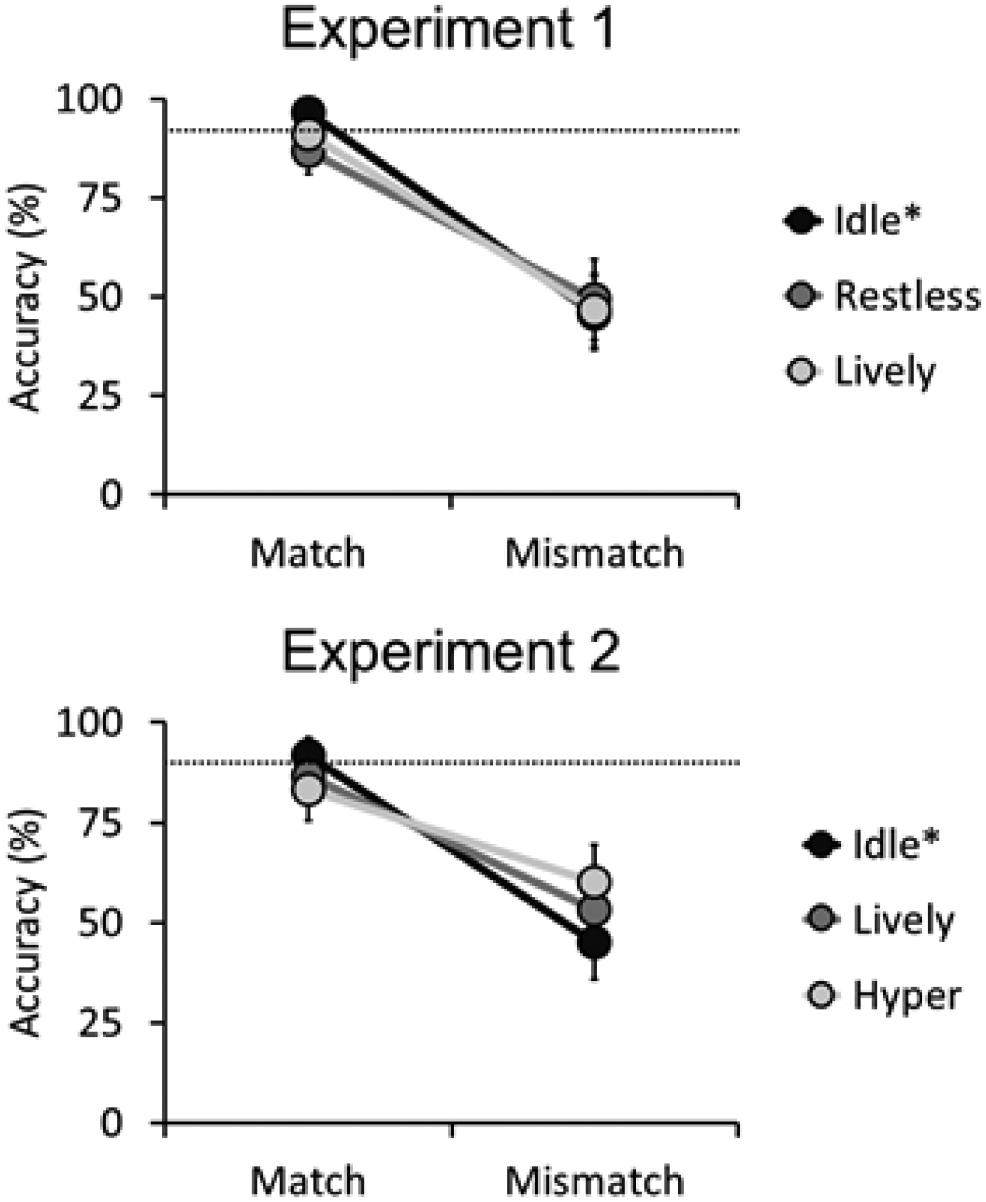
Mean accuracy scores of the critical match and mismatch trials for each activity level and trial type for Experiments 1 and 2. The error bars represent the standard errors of the means. The dashed line represents the mean accuracy for the noncritical match trials, the activity level of which is denoted by the asterisk (i.e., the majority activity level).

Overall accuracy for the 88 noncritical match trials was 92.3% (*SD* = 5.2). Inferential analysis was applied only to the data for the six critical match and six mismatch trials, which are displayed in Figure 2. In a first step of this analysis, participants were given an awareness score of how far their reported number of activity levels deviated from the actual number. For example, those who correctly reported three levels scored 0, while those who reported only one level scored –2. Of the 30 participants, 12 correctly reported that there were three activity levels. This body language awareness score was used as a covariate in the inferential analysis. The critical data were then analysed using a 2 (trial type [match vs. mismatch]) × 3 (activity level [idle vs. restless vs. lively]) within-subjects analysis of covariance.^1^ This showed a main effect of trial type, *F*(1, 28) = 43.21, *p* < .001, η_p_^2^ = .61, due to higher match than mismatch accuracy, but no main effect of activity level, *F*(2, 56) = 0.34, *p* = .72, η_p_^2^ = .01, nor awareness, *F*(1, 28) = 1.45, *p* = .24, η_p_^2^ = .04. Two-way interactions between any of the factors, all *Fs* ≤ 1.01, all *ps* ≥ .37, all η_p_^2^ ≤ .04, and a three-way interaction were not found, *F*(2, 56) = 0.97, *p* = .39, η_p_^2^ = .03.

#### d-prime and Criterion

The accuracy data were also converted to *d′* and *criterion* to examine sensitivity and response bias. One-factor analyses of variance (ANOVAs) did not show an effect of activity level for *d′* (idle = 0.40, restless = 0.37, lively = 0.35), *F*(2, 58) = 0.12, *p* = .88, η_p_^2^ < .01, or *criterion* (idle = –0.35, restless = –0.21, lively = –0.29), *F*(2, 58) = 1.84, *p* = .17, η_p_^2^ = .06. These results therefore converge with the analysis of the percentage accuracy data to show that body language did not affect face matching.

### Discussion

This experiment manipulated the activity levels of avatars within a virtual passport control environment to examine whether body language influences face-matching decisions. Identity matches with idle body language presented the majority of trials, and their classification was near ceiling. By comparison, the occurrence of identity mismatches was low, reflecting only 6% of trials, and these face pairs were only classified correctly in half of these. Importantly to the question of main interest, however, classification of matches and mismatches was not affected by variation in body activity levels.

This pattern of results was observed irrespective of whether participants reported awareness of the differences in body language. However, participants were not made aware of this manipulation prior to the experiment, and only 12 of the 30 participants were accurate in reporting that three different activity levels of body language were used. Thus, it is possible that the body language manipulation that was applied here was too weak to be detected consistently by observers and, therefore, to influence person identification.

## Experiment 2

In the previous experiment, the activity level of body language did not influence person identification. However, most participants showed limited awareness of the body language manipulation. It is therefore possible that differences in body language were too subtle to elicit an effect. To investigate this possibility, the different body language activity levels were increased in Experiment 2, to exaggerate the perceptual differences between conditions. For this purpose, the idle and lively activity levels of Experiment 1 were retained, but a new hyper condition, in which the lively activity level was doubled in magnitude, was added.

### Method

#### Participants

Thirty Caucasian students from the University of Kent (7 male, 23 female), with a mean age of 21.0 years (*SD* = 6.7 years), participated in exchange for course credit. All participants reported normal or corrected-to-normal vision, and none had participated in Experiment 1.

#### Stimuli and Procedure

The stimuli and procedure were identical to the preceding experiment except for the scale factors of the animation. The variations in animation cycle duration were increased to enable the higher activity trials to appear more perceptually different to the idle activity trials (Scale Factor 1, animation cycle of 13.3 seconds). The lively activity trials were retained (Scale Factor 3, cycle of 4.4 seconds), but hyper activity trials (Scale Factor 6, cycle of 2.2 seconds) replaced the restless trials (Scale Factor 2, cycle of 6.7 seconds).

### Results

#### Percentage Accuracy

The data for this experiment were analysed using the same method as Experiment 1. Overall accuracy for the 88 noncritical match trials was 90.4% (*SD* = 8.2). Inferential analysis was applied to the data for the six critical match and six mismatch trials, the data that can be seen in Figure 2. An awareness score was again calculated based on participants’ questionnaire responses. Of the 30 participants, 15 correctly reported that there were three activity levels. A 2 (trial type [match vs. mismatch]) × 3 (activity level [idle vs. lively vs. hyper]) within-subjects analysis of covariance with the covariate of awareness showed a main effect of trial type, *F*(1, 28) = 26.53, *p* < .001, η_p_^2^ = .49, due to match trials being classified more accurately than mismatch trials. However, there was no main effect of activity level, *F*(2, 56) = 0.02, *p* = .98, η_p_^2^ < .01, or awareness, *F*(1, 28) = 1.02, *p* = .32, η_p_^2^ = .04, no two-way interactions between any of the factors, all *Fs* ≤ 1.87, all *ps* ≥ .16, all η_p_^2^ ≤ .06, and no three-way interaction, *F*(2, 56) = 0.36, *p* = .70, η_p_^2^ = .01.

#### d-prime and Criterion

The accuracy data were also converted to *d′* and *criterion* to examine sensitivity and response bias. As in Experiment 1, one-factor ANOVAs did not show an effect of activity level for *d′* (idle = 0.35, lively = 0.38, hyper = 0.41), *F*(2, 58) = 0.21, *p* = .82, η_p_^2^ = .01, or *criterion* (idle = –0.31, lively = –0.22, hyper = –0.16), *F*(2, 58) = 2.75, *p* = .07, η_p_^2^ = .09.

### Discussion

This experiment replicates the results of Experiment 1 closely. Match accuracy was higher than mismatch accuracy, and around half of the participants reported awareness of the three body activity levels. However, despite the increase in body language activity, this did not influence face-matching accuracy. This finding appears at odds with previous literature suggesting that body cues, even when processed unconsciously, affect facial identification (Rice, Phillips, Natu, et al., 2013), but this research relied on identity information from the body rather than body *activity*. This contrast suggests that, if body language affects person identification in the VR airport paradigm, then this may require conscious monitoring of such behavioural cues to have an impact on face-matching decisions.

## Experiment 3

In Experiments 1 and 2, matching avatar faces to their passport image was the primary task, and participants were not required to monitor body language closely, which may explain why this did not influence identification decisions. In Experiment 3, participants were therefore instructed directly to monitor variation in body language, with a view to aiding the detection of mismatches, to determine whether such explicit instruction is required to influence identification decisions.

### Method

#### Participants

A further 30 Caucasian participants (8 male, 22 female), with a mean age of 19.3 years (*SD* = 1.3 years), were recruited from the University of Kent for course credit. All participants reported normal or corrected-to-normal vision. None had participated in the previous experiments.

#### Stimuli and Procedure

For this experiment, participants were informed about the animation of the avatars. It was explained prior to the task that the avatars would be shifting in their stance while waiting, that this level of activity could vary, and that such differences in body language might be useful for detecting identity mismatches.^2^ Owing to the inclusion of this additional instruction, participants did not complete the questionnaire for reporting avatar animation from Experiments 1 and 2. All other aspects of the stimuli and procedure remained identical. Thus, most avatars comprised of identity matches displaying idle behaviour, with a subset of six critical matches and six mismatches displaying idle, lively, and hyper body language.

### Results

#### Percentage Accuracy

**Figure 3.**
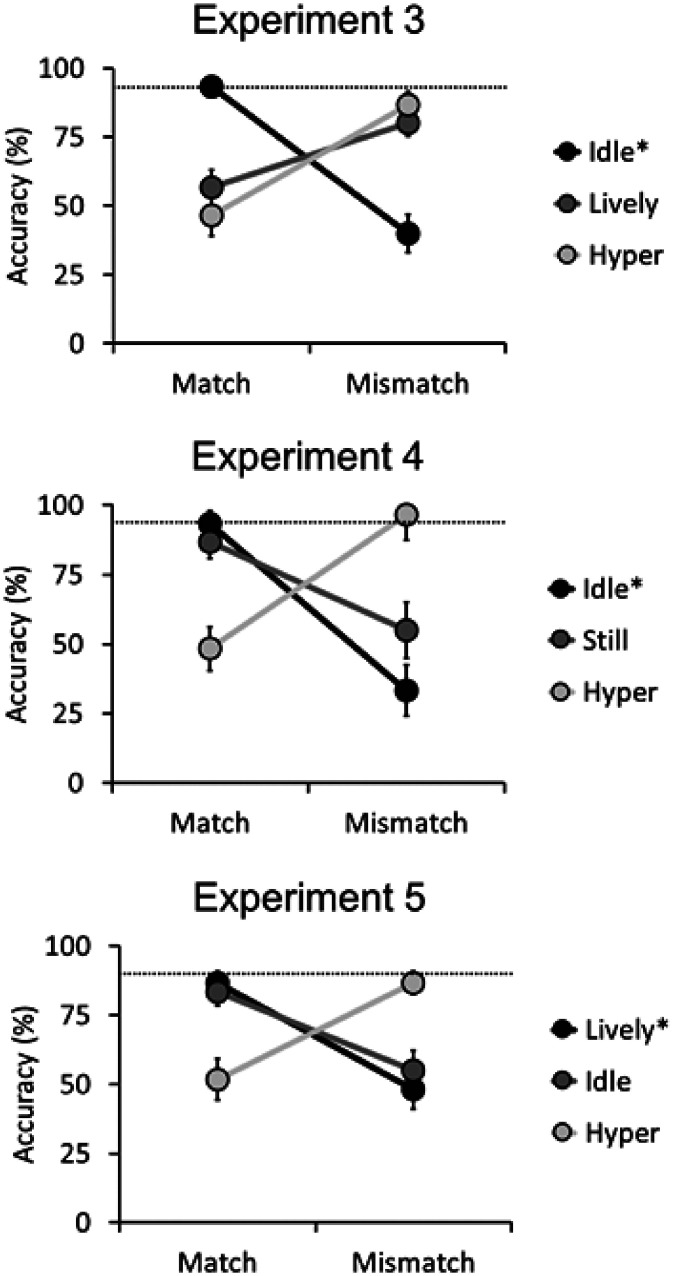
Mean accuracy scores of the critical match and mismatch trials for each activity level and trial type for Experiments 3, 4, and 5. The error bars represent the standard errors of the means. The dashed line represents the mean accuracy for the noncritical match trials, the activity level of which is denoted by the asterisk (i.e., the majority activity level).

Overall accuracy for the 88 noncritical match trials was 92.8% (*SD* = 5.5). Mean accuracy for the six critical match and mismatch trials are displayed in Figure 3. A 2 (trial type [match vs. mismatch]) × 3 (activity level [idle vs. lively vs. hyper]) within-subjects ANOVA of these data did not show a main effect of trial type, *F*(1, 29) = 0.66, *p* = .42, η_p_^2^ = .02, or activity level, *F*(2, 58) = 0.08, *p* = .92, η_p_^2^ < .01, but an interaction of these factors, *F*(2, 58) = 32.83, *p* < .001, η_p_^2^ = .53.

Analysis of simple main effects revealed an effect of activity level for match trials, *F*(2, 28) = 21.40, *p* < .001, η_p_^2^ = .60, with paired-samples *t* tests (with alpha corrected to .017 [.05/3] for three comparisons) showing that idle match trials were identified more accurately than both lively and hyper match trials, *t*(29) = 5.81, *p* < .001, *d* = 1.34 and *t*(29) = 5.22, *p* < .001, *d* = 1.45, respectively. Accuracy for lively and hyper match trials did not differ, *t*(29) = 1.14, *p* = .26, *d* = 0.26.

A simple main effect of activity level for mismatch trials was also found, *F*(2, 28) = 19.22, *p* < .001, η_p_^2^ = .58. Paired-samples *t* tests (with alpha corrected to .017 [.05/3] for three comparisons) showed that both lively and hyper mismatch trials were identified more accurately than idle mismatch trials, *t*(29) = 4.94, *p* < .001, *d* = 1.23 and *t*(29) = 6.18, *p* < .001, *d* = 1.41, respectively. As with match trials, accuracy was comparable for lively and hyper mismatch trials, *t*(29) = 1.00, *p* = .33, *d* = 0.26.

Finally, a simple main effect of trial type was found within the idle activity level, *F*(1, 29) = 49.83, *p* < .001, η_p_^2^ = .63, with match trials being performed more accurately than mismatch trials. By contrast, mismatch accuracy was higher than match accuracy within the lively, *F*(1, 29) = 8.83, *p* < .01, η_p_^2^ = .23, and hyper activity level, *F*(1, 29) = 19.33, *p* < .001, η_p_^2^ = .40.

#### d-prime and Criterion

One-factor ANOVAs did not show an effect of activity level for *d′* (idle = 0.32, lively = 0.35, hyper = 0.30), *F*(2, 58) = 0.14, *p* = .87, η_p_^2^ = .01, but for *criterion* (idle = –0.36, lively = 0.16, hyper = 0.28), *F*(2, 58) = 32.51, *p* < .001, η_p_^2^ = .53. Paired-samples *t* tests (with alpha corrected to .017 [.05/3] for three comparisons) revealed a mismatch bias on hyper and lively trials compared with idle trials, *t*(29) = 6.76, *p* < .001, *d* = 2.03 and *t*(29) = 6.86, *p* < .001, *d* = 1.80, respectively. Criterion for hyper and lively trials did not differ, *t*(29) = 1.52, *p* = .14, *d* = 0.37.

### Discussion

In this experiment, participants were informed that differences in body language might be useful for the detection of mismatches, to investigate whether this influences person identification when observers are explicitly instructed to monitor for such cues. In contrast to Experiments 1 and 2, body language exerted a clear effect on the classification of identity matches and mismatches. Matches exhibiting idle body language were detected with near-perfect accuracy, whereas more than half of all mismatches with this body language were classified incorrectly. By contrast, identification of lively and hyper matches was reduced greatly, whereas mismatch classification was near ceiling in these body language conditions. These findings indicate that observers employed unusual body language as a heuristic to support mismatch decisions. However, rather than serving to highlight passengers exhibiting unusual body language to enhance scrutiny of their facial identity, which might increase accuracy on both match and mismatch trials, unusual body language appeared to *bias* the classification of all face pairs towards mismatch classification.

## Experiment 4

The results of Experiment 3 indicate that avatars exhibiting unusual body language increased mismatch classifications, both of identity matches and mismatches. However, unusual body language was always characterised by raising activity levels from idle to lively and hyper in Experiment 3. Therefore, the question arises of whether this effect is driven by an *increase* in normal body language, or reflects that lively and hyper avatars are behaving *differently* to the majority of idle avatars in the experiment.

This is an interesting question to consider also in the context of the psychological literature on the detection of deception. While laypersons and professionals, such as customs and police officers, tend to believe that increases in body language are indicators of deception (e.g., Akehurst et al., 1996; Bogaard et al., 2016; Kraut & Poe, 1980; Masip & Herrero, 2015; Vrij & Semin, 1996), deceptive behaviour may be characterised *actually* by reduced body movements (Akehurst et al., 1996; Vrij, 2008a; Vrij & Mann, 2001; for reviews, see DePaulo & Morris, 2004; Strömwall et al., 2004). Considering that identity mismatches reflect a form of deception, the question therefore arises of whether the detection of these face pairings is affected both by increases and *decreases* in body language relative to the norm.

To investigate this issue, the majority of avatars again displayed idle body language in Experiment 4 and a subset exhibited hyper activity levels. However, the lively condition was replaced with still avatars, which displayed no movement during identification. If the increase in mismatch decisions that was observed in Experiment 3 is driven by nonverbal behaviour that is unusual from the norm, then this effect should be observed with both still and hyper avatars in Experiment 4. If, on the other hand, this effect relies on increased body language, then it should be observed only with hyper avatars.

### Method

#### Participants

Thirty Caucasian participants (9 male, 21 female), with a mean age of 19.9 years (*SD* = 2.7 years), who had not participated in any of the previous experiments, were recruited from the University of Kent. All reported normal or corrected-to-normal vision and were granted course credit or a small fee for their participation.

#### Stimuli and Procedure

The stimuli and procedure were identical to the previous experiment except for changes to the animation cycles. Idle and hyper activity levels maintained scale factors of 1 and 6 (i.e., with animation cycles of 13.3 and 2.2 seconds, respectively), but lively trials were replaced with a still condition with a scale factor of 0 (i.e., no animation cycle). Thus, avatars in the still condition approached the passport control desk and then did not move at all during identification.

### Results

#### Percentage Accuracy

Overall accuracy for the 88 noncritical match trials was 94.0% (*SD* = 4.3). Mean accuracy rates for the six critical match and mismatch trials are displayed in Figure 3. A 2 (trial type [match vs. mismatch]) × 3 (activity level [still vs. idle vs. hyper]) within-subjects ANOVA of these data did not show a main effect of activity level, *F*(2, 58) = 2.19, *p* = .12, η_p_^2^ = .07, but a main effect of trial type, *F*(1, 29) = 8.44, *p* < .01, η_p_^2^ = .23, and an interaction between factors, *F*(2, 58) = 48.92, *p* < .001, η_p_^2^ = .63.

Analysis of simple main effects revealed an effect of activity level for match trials, *F*(2, 28) = 17.81, *p* < .001, η_p_^2^ = .56, with paired-samples *t* tests (with alpha corrected to .017 [.05/3] for three comparisons) showing that idle and still match trials were performed more accurately than hyper match trials, *t*(29) = 5.84, *p* < .001, *d* = 1.50 and *t*(29) = 5.14, *p* < .001, *d* = 1.16, respectively. Accuracy for idle and still match trials did not differ, *t*(29) = 1.07, *p* = .29, *d* = 0.30.

A simple main effect of activity level within mismatch trials was also found, *F*(2, 28) = 43.77, *p* < .001, η_p_^2^ = .76, with paired-samples *t* tests (with alpha corrected to .017 [.05/3] for three comparisons) showing higher accuracy for hyper mismatches than idle, *t*(29) = 8.38, *p* < .001, *d* = 2.21, and still mismatches, *t*(29) = 6.53, *p* < .001, *d* = 1.54. In addition, accuracy on still mismatch trials was also higher than on idle mismatch trials, *t*(29) = 2.54, *p* < .017, *d* = 0.58.

Finally, simple main effects of trial type were found within the idle, *F*(1, 29) = 60.23, *p* < .001, η_p_^2^ = .68, and still activity levels, *F*(1, 29) = 12.94, *p* < .001, η_p_^2^ = .31, due to higher accuracy for match than mismatch trials. Within the hyper activity level, the reverse pattern was observed, with higher accuracy on mismatch than match trials, *F*(1, 29) = 35.40, *p* < .001, η_p_^2^ = .55.

#### d-prime and Criterion

One-factor ANOVAs did not show an effect of activity level for *d′* (still = 0.40, idle = 0.25, hyper = 0.43), *F*(2, 58) = 2.19, *p* = .12, η_p_^2^ = .07, but for *criterion* (still = –0.21, idle = –0.40, hyper = 0.33), *F*(2, 58) = 48.92, *p* < .001, η_p_^2^ = .63. Paired-samples *t* tests (with alpha corrected to .017 [.05/3] for three comparisons) revealed a greater mismatch bias on hyper trials compared with idle, *t*(29) = 8.85, *p* < .001, *d* = 2.44, and still trials, *t*(29) = 7.74, *p* < .001, *d* = 1.69. There was no difference in *criterion* between idle and still trials after correcting for multiple comparisons, *t*(29) = 2.48, *p* = .019, *d* = 0.60.

### Discussion

This experiment replicates the key aspects of Experiment 3, by showing that match accuracy declined and mismatch accuracy was enhanced when avatars display unusually hyper body language. The current experiment examined in addition whether a similar bias is found when avatars do not display any body movement during identification. On match trials, accuracy for idle and still avatars converged. Thus, in a context in which the majority of avatars display idle body language, the absence of body language did not influence classification of the identity matches. On mismatch trials, on the other hand, still avatars were classified correctly more often than idle avatars, but this effect was much smaller than for hyper avatars. Overall, these findings suggest that it is predominantly an *increase* in body language, rather than unusual body language, which affects face identification in the VR airport paradigm here. However, an alternative explanation is also possible, as the scale factors for still and idle avatars were more closely matched (at 0 and 1, respectively) than for hyper avatars (6). This opens the possibility that performance for still and idle avatars was more comparable due to the greater perceptual similarity of these activity levels, in comparison with hyper trials.

## Experiment 5

In Experiment 4, still avatars exerted a much more limited influence on facial identification than hyper avatars, which suggests that it is an increase in body language, rather than unusual body language, which determines these effects. However, the activity levels of still and idle avatars were also more closely matched relative to the hyper condition. To investigate whether this can account for the results of Experiment 4, we conducted another experiment in which the noncritical avatars now exhibited lively instead of idle body language, with critical matches and mismatches exhibiting idle, lively, and hyper activity levels. In contrast to the preceding experiments, the majority of avatars therefore exhibited lively body language, and hyper *as well as* idle movement represented the unusual body language conditions. Thus, this experiment provides a better test for whether the effect of body language in the preceding experiments is due to some avatars exhibiting *increased* or *unusual* body language (i.e., increased or decreased activity levels from the norm).

### Method

#### Participants

Thirty Caucasian participants (4 male, 26 female), with a mean age of 22.2 years (*SD* = 5.4 years), were recruited from the University of Kent and granted course credit or a small fee for their participation. None of these individuals had participated in the previous experiments. All reported normal or corrected-to-normal vision.

#### Stimuli and Procedure

The method and procedure were identical to Experiment 3 with the exception that the noncritical match trials now displayed the lively activity level. As a result, most avatars displayed lively behaviour (at Scale Factor 3), with a small subset of avatars displaying idle (low activity; Scale Factor 1) or hyper (high activity; Scale Factor 6) behaviour.

### Results

#### Percentage Accuracy

Overall accuracy for the 88 noncritical match trials was 89.6% (*SD* = 10.1). Mean accuracy scores were calculated for the six critical match and mismatch trials and are displayed in Figure 3. A 2 (trial type [match vs. mismatch]) × 3 (activity level [idle vs. lively vs. hyper]) within-subjects ANOVA of this data did not show main effects of trial type, *F*(1, 29) = 2.89, *p* = .10, η_p_^2^ = .09, or activity level, *F*(2, 58) = 0.07, *p* = .94, η_p_^2^ < .01, but an interaction between these factors, *F*(2, 58) = 20.12, *p* < .001, η_p_^2^ = .41.

For match trials, a simple main effect of activity level was found, *F*(2, 28) = 7.52, *p* < .01, η_p_^2^ = .35, with paired-samples *t* tests (with alpha corrected to .017 [.05/3] for three comparisons) showing that lively and idle matches were classified more accurately than hyper matches, *t*(29) = 3.88, *p* < .001, *d* = 1.06 and *t*(29) = 3.60, *p* < .001, *d* = 0.91, respectively. In contrast, accuracy was comparable for lively and idle match trials, *t*(29) = 0.63, *p* = .57, *d* = 0.13.

A corresponding simple main effect of activity level was found for mismatch trials, *F*(2, 28) = 16.27, *p* < .001, η_p_^2^ = .54, due to the more accurate classification of hyper than lively, *t*(29) = 4.89, *p* < .001, *d* = 1.16, and idle mismatches, *t*(29) = 4.08, *p* < .001, *d* = 0.96. Also as for identity matches, accuracy for lively and idle mismatches did not differ, *t*(29) = 0.68, *p* = .50, *d* = 0.16.

Finally, simple main effects of trial type within the lively, *F*(1, 29) = 17.41, *p* < .001, η_p_^2^ = .38, and idle conditions were found, *F*(1, 29) = 7.90, *p* < .01, η_p_^2^ = .21, due to higher accuracy for match than mismatch trials. For the hyper condition, the reverse pattern of superior mismatch accuracy was shown, *F*(1, 29) = 14.07, *p* < .001, η_p_^2^ = .33.

#### d-prime and Criterion

One-factor ANOVAs did not show an effect of activity level for *d′* (idle = 0.37, lively = 0.33, hyper = 0.37), *F*(2, 58) = 0.07, *p* = .94, η_p_^2^ < .01, but for *criterion* (idle = –0.19, lively = –0.26, hyper = 0.24), *F*(2, 58) = 20.12, *p* < .001, η_p_^2^ = .41, due to a greater mismatch bias on hyper compared with lively and idle trials, *t*(29) = 6.03, *p* < .001, *d* = 1.45 and *t*(29) = 5.09, *p* < .001, *d* = 1.19, respectively. *Criterion* for lively and idle trials did not differ, *t*(29) = 0.77, *p* = .45, *d* = 0.19.

### Discussion

As in Experiments 3 and 4, accuracy for trials with the most common activity level, which was lively body language in this case, was high when these were identity matches and low when these were mismatches. This demonstrates that mismatches are frequently missed in this paradigm when nonverbal cues from unusual body language are not available. Once again, this pattern was reversed dramatically when such unusually hyper body language was exhibited. The primary aim of this experiment was to confirm whether these effects are present only when unusual body language is characterised by an increase in activity compared with the norm, or also when activity levels are attenuated on idle trials. Classification of idle matches and mismatches aligned with the most common lively condition. This provides converging evidence with Experiment 4 to indicate that the current effects are driven by increased expressive body language rather than body language that differs from the norm per se.

## Experiment 6

The experiments reported so far demonstrate that unusual body language strongly biases face-matching decisions. In a final experiment, we explore why this identification bias might arise. One possibility is that observers adopt unusual body language as a simple heuristic to identify mismatches, in turn reducing reliance on the available facial information. Such a heuristic would not be available for trials on which the body language reflects that of the majority of avatars, which should therefore rely to a greater extent on facial information. It is possible to investigate this possibility by comparing performance on the VR passport task with established laboratory tests of face matching ability, such as the Glasgow Face Matching Test (GFMT; Burton et al., 2010) and the Kent Face Matching Test (KFMT; Fysh & Bindemann, 2018b). These tests correlate with a range of facial discrimination and identification tasks (e.g., Fysh, 2018; Fysh & Bindemann, 2018b; McCaffery et al., 2018), including the identity matching of the avatars that were employed in the current experiments (Tummon et al., 2019). This indicates that these face tests present suitable and stable measures against which person identification in the VR task can be compared.

In Experiment 6, we employ two activity level conditions, whereby the majority of avatars exhibit idle body language and the unusual body language condition comprises of lively avatars. Match and mismatch accuracy for these activity levels is then correlated with performance on the GFMT and KFMT. Consistent with previous research (Tummon et al., 2019), identification of idle avatars should correlate with matching performance on the GFMT and KFMT in this design. Conversely, if unusual body language reduces observers’ reliance on facial information of the avatars, then identification performance on lively trials should correlate to a lesser extent with accuracy on the GFMT and KFMT.

### Method

#### Participants

One-hundred Caucasian students from the University of Kent (18 male, 82 female), with a mean age of 19.3 years (*SD* = 1.8 years), participated in this experiment in exchange for course credit. All participants reported normal or corrected-to-normal vision. None had participated in any of the previous experiments.

#### Stimuli and Procedure

For this experiment, the following modifications were made to the VR passport control task. The same proportion of 88 noncritical match trials, 6 critical match trials, and 6 mismatch trials was used. However, only two activity level conditions were employed, comprising idle and lively body language, resulting in the presentation of three match and mismatch trials of each. In addition, the order of the 100 trials was fixed, with critical matches displayed on Trial 18, 35, 61, 66, 87, and 92, and mismatches on Trial 24, 28, 48, 71, 83, and 97. These 12 trials alternated in activity level condition, which was counterbalanced across participants. These changes were implemented to ensure that performance was more directly comparable across observers for the correlational analysis. All remaining aspects of this task remained the same as in Experiments 3 to 5, with participants instructed a priori about the animation conditions of the avatars.

In addition to the VR passport control task, the GFMT (Burton et al., 2010) and KFMT (Fysh & Bindemann, 2018b) were included as additional tasks in this experiment. The GFMT face pairs consist of images of faces taken from a frontal view displaying a neutral expression. Both images in a face pair are taken with different cameras and, in the case of identity matches, approximately 15 minutes apart. Each face image is cropped to show the head only and converted to greyscale with a resolution of 72 ppi. The dimensions of the faces range in width from 70 mm to 90 mm and in height from 85 mm to 125 mm and are spaced between 40 mm and 55 mm apart on screen. This experiment employed 20 identity match and 20 mismatch trials from the GFMT (for more information, see Burton et al., 2010).

**Figure 4.**
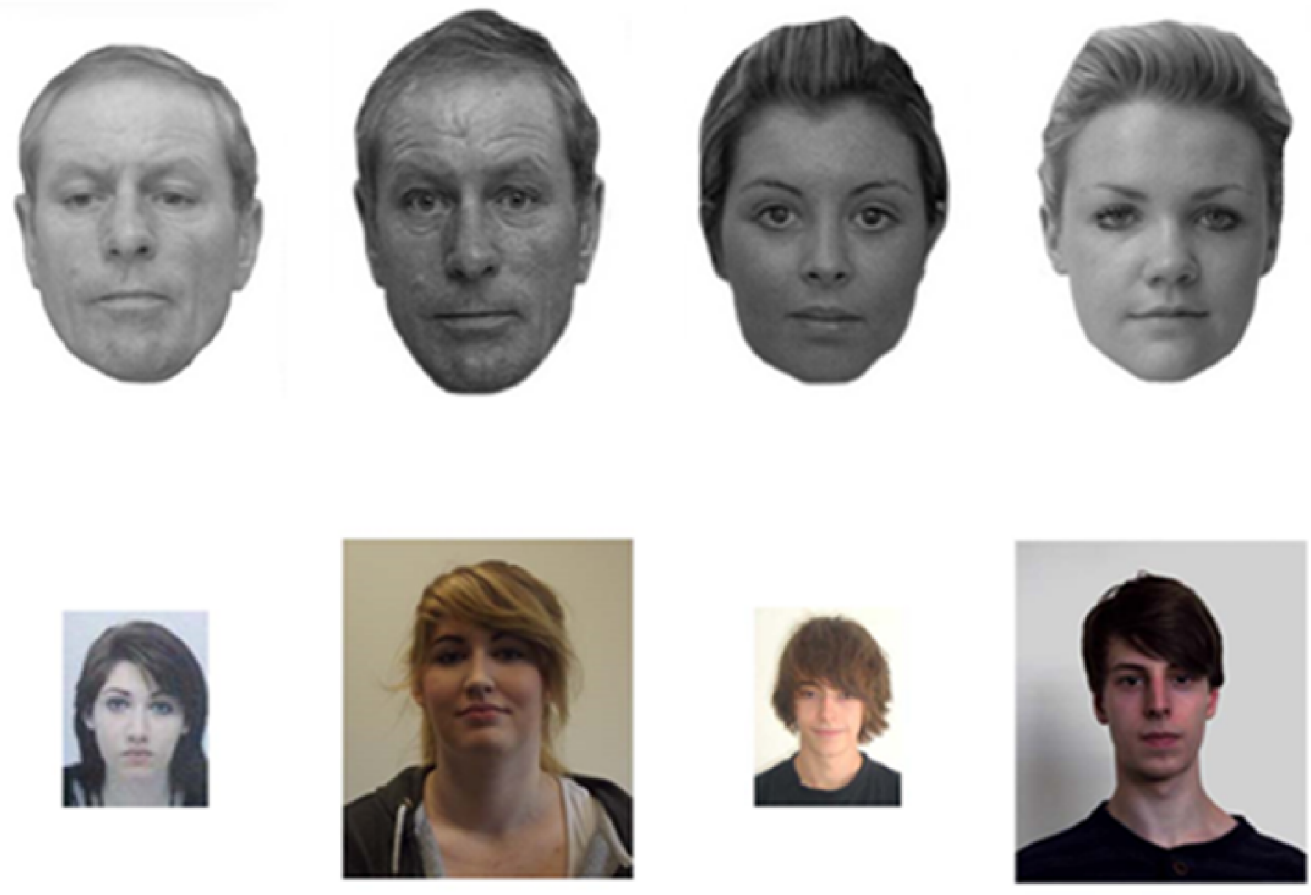
Example stimuli of match (left) and mismatch (right) trials for the GFMT (top row) and KFMT (bottom row).

The KFMT face pairs consist of an image from a student ID card, presented at a maximal size of 35 mm (w) × 47 mm (h), and a portrait photo, sized at 70 mm (w) × 82 mm (h) at a resolution of 72 ppi, spaced 75 mm apart. The student ID photos were taken at least 3 months prior to the face portraits and were not constrained by pose, facial expression, or image-capture device. The portrait photos depict the target’s head and shoulders from a frontal view while bearing a neutral facial expression and were captured with a high-quality digital camera. In this experiment, 20 identity match and 20 mismatch trials from the KFMT were employed (for more information, see Fysh & Bindemann, 2018b). Example stimuli for the two face matching tests are shown in Figure 4. The GFMT and KFMT tasks were presented using PsychoPy (Peirce, 2007) and were completed after the VR passport control task, with the order counterbalanced across participants.

### Results

#### GFMT and KFMT Performance

**Figure 5.**
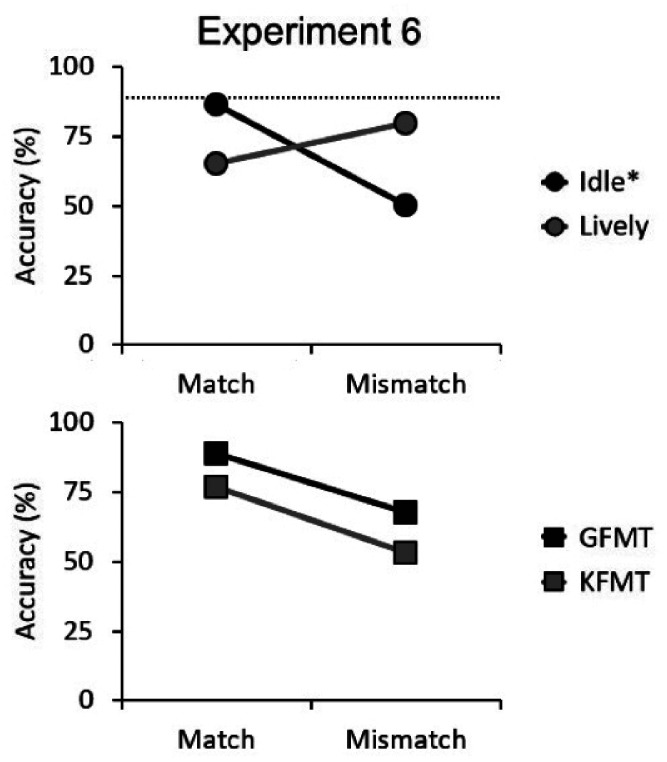
Mean accuracy scores of the critical match and mismatch trials for the idle and lively activity levels in the VR passport control task (circles) and match and mismatch accuracy on the two face-matching tests (squares) for Experiment 6. The standard errors of the means are too small (ranging from 1.1 to 3.3) for the error bars to be visible. The dashed line represents the mean accuracy for the noncritical match trials on the VR passport control task, the activity level of which is denoted by the asterisk (i.e., the majority activity level). GFMT = Glasgow Face Matching Test; KFMT = Kent Face Matching Test.

The mean percentage accuracy on the GFMT and KFMT is illustrated in Figure 5. To establish that performance in the face-matching tests conformed with previous findings, a 2 (trial type [match vs. mismatch]) × 2 (face-matching task [GFMT vs. KFMT]) within-subjects ANOVA was conducted. Consistent with previous work (Fysh & Bindemann, 2018b), this showed a main effect of test, *F*(1, 99) = 217.58, *p* < .001, η_p_^2^ = .69, whereby the GFMT was performed more accurately than the KFMT. A main effect of trial type was also found, *F*(1, 99) = 90.32, *p* < .001, η_p_^2^ = .48, due to higher match than mismatch accuracy. There was no interaction between these factors, *F*(1, 99) = 1.34, *p* = .25, η_p_^2^ = .01.

Correspondingly, a paired-samples *t* test revealed that *d′* was higher for the GFMT (1.44) than the KFMT (0.67), *t*(99) = 14.50, *p* < .001, *d* = 1.38. In addition, a second paired-samples *t* test for *criterion* showed a greater bias to make match responses on the GFMT (–0.45) than the KFMT (–0.36), *t*(99) = 2.13, *p* < .05, *d* = 0.19.

#### VR Performance

In the next step of the analysis, we sought to confirm the body language effect that was observed in Experiments 3 to 5. Accuracy for the 88 noncritical match trials on the VR passport control task was 89.0% (*SD* = 9.2). Mean accuracy for the six critical match and mismatch trials is displayed in Figure 5. A 2 (trial type [match vs. mismatch]) × 2 (activity level [idle vs. lively]) within-subjects ANOVA of this data did not show a main effect of activity level, *F*(1, 99) = 3.24, *p* = .08, η_p_^2^ = .03, but a main effect of trial type, *F*(1, 99) = 9.28, *p* < .01, η_p_^2^ = .09, and an interaction between factors, *F*(1, 99) = 78.58, *p* < .001, η_p_^2^ = .44.

Analysis of simple main effects revealed an effect of activity level for match trials, *F*(1, 99) = 35.25, *p* < .001, η_p_^2^ = .26, reflecting that idle trials were classified more accurately than lively trials. A simple main effect of activity level was also found for mismatch trials, *F*(1, 99) = 61.37, *p* < .001, η_p_^2^ = .38, but due to superior accuracy for lively trials. In addition, simple main effects of trial type were revealed within both the idle and the lively activity levels, *F*(1, 99) = 66.01, *p* < .001, η_p_^2^ = .40 and *F*(1, 99) = 9.85, *p* < .01, η_p_^2^ = .09, respectively. For the idle activity level, this was due to higher accuracy on match trials than mismatch trials, while the reverse pattern was observed for the lively activity level.

Finally, a paired-samples *t* test showed that *d′* did not differ for the idle (0.44) and lively (0.53) activity levels, *t*(99) = 1.71, *p* = .09, *d* = 0.22. However, *criterion* revealed a greater bias to make mismatch responses on lively (0.13) than idle trials (–0.30), *t*(99) = 8.89, *p* < .001, *d* = 1.13. Overall, these findings therefore converge with previous experiments to show that unusual body language biases observers’ responses, resulting in an increase in mismatch decisions on lively body language trials.

#### Correlational Analyses Between Tasks

In the final step of this analysis, a series of Pearson correlations was carried out to assess how performance on the VR passport control task relates to individual differences in face-matching performance. Consistent with previous research, accuracy on the GFMT and KFMT correlated both for match, *r* = .529, *p* < .001, and mismatch trials, *r* = .718, *p* < .001. Performance on these two face-matching tests was then compared with the VR passport control task, both under idle and lively body language conditions. Accuracy on idle match trials correlated with match accuracy on the GFMT, *r* = .280, *p* < .01, but not the KFMT, *r* = .149, *p* = .14. For mismatch trials, accuracy under idle body language conditions correlated with both tests, *r* = .232, *p* < .05 and, *r* = .234, *p* < .05, respectively. In contrast to idle body language, accuracy on lively match trials did not correlate with GMFT, *r* = .101, *p* = .32, or KFMT match accuracy, *r* = .141, *p* = .16. These findings extend to the mismatch conditions, for which accuracy on lively trials also did not correlate with the GFMT, *r* = .131, *p* = .19, or KFMT, *r* = .112, *p* = .27.

### Discussion

This experiment examined whether the biasing effect of body language reflects a reduced reliance on facial information. To investigate this possibility, the VR passport control task was modified to consist of frequently occurring idle and infrequently occurring lively activity level trials. Performance on this task was then compared with two established face matching tests, comprising the GFMT (Burton et al., 2010) and KFMT (Fysh & Bindemann, 2018b).

Overall, the VR experiment replicated the body language bias of the preceding experiments, by demonstrating that observers were more likely to classify face pairs as identity mismatches when these exhibited unusually lively body language, in comparison with the frequent idle body language trials. Consistent with previous research, the GFMT also proved to be an easier face-matching task than the KFMT, and accuracy across both of these tests correlated well (Fysh & Bindemann, 2018b). More importantly, individual performance on the face matching tests also correlated with the VR task. This finding is consistent with a previous validation of this VR paradigm, in which similar correlations were reported between the GFMT, KFMT, and the matching of avatar faces (Tummon et al., 2019). In Experiment 6, however, such correlations were observed only for idle trials. For lively trials, no association between GFMT, KFMT, and identification accuracy in VR was found.

This combination of results indicates that related processes are employed to complete the two face-matching tests *and* the VR passport control task when body language is normal. When unusually lively body language is displayed, on the other hand, this information dominates decision-making during identification to the extent that this process no longer associates with face identification ability. This suggests that, rather than highlighting travellers’ faces for particular scrutiny during passport control, unusual body language undermines person identification in this environment by reducing reliance on the primary facial identity cues.

## General Discussion

This study employed a novel VR paradigm to investigate whether nonverbal cues from body language influence person identification. Participants assumed the role of passport control officers in a VR airport and were required to process a queue of passengers by comparing their faces to passport-style photo cards to detect identity mismatches. We manipulated the body language of these mismatches and a corresponding set of matches so that this either reflected the behaviour of the majority of passengers or was more or less active than this norm. When participants were not informed in advance of this variation in body language in Experiments 1 and 2, this manipulation did not influence person identification. In contrast, when participants were explicitly instructed to monitor variation in body language to aid classification of mismatches in Experiments 3 to 6, the detection of these cases increased. The magnitude of this effect was substantial, resulting in an increase in mismatch detections of nearly 50% across experiments when body language was unusually lively or hyper.

The finding that body language affects only person identification with conscious monitoring deviates from previous work, which suggests that the body influences identification even when observers have limited awareness of this (Rice, Phillips, Natu, et al., 2013). In contrast to this previous research, however, body cues could not be used directly for identification in the current study, as only the avatars' faces were visible on the photo-identity documents (see Figure 1). Rather, in the current study, body language served to alert observers to the presence of potential identity mismatches so that more care could be given to the accurate facial identification of these infrequent trial types. This difference between studies may explain why body language needed to be actively monitored in the experiments here, to exert an effect on the separable task of face matching.

Another reason as to why the body language effect hinged on explicit instruction to look for these cues may be that, without some prior warning, it may not be clear what unusual body language is. In the current study, the effect of body language was observed consistently when the activity level of this behaviour was higher than the norm. In contrast, when body language differed from the norm through reduced activity, this caused only a small (Experiment 4) or no increase (Experiment 5) in mismatch accuracy. This indicates that it was not unusual body language per se that influenced mismatch detection, but specifically an increase in behavioural activity. This effect is particularly striking in a comparison of Experiments 3 and 5. Both experiments included idle, lively, and hyper body language, and participants were given identical instructions, but the experiments differed in terms of which of the body activity levels was assigned as the norm (idle in Experiment 3, lively in Experiment 5). When unusual body language was defined as increased activity from this norm in Experiment 3, both of the unusual body language conditions affected face matching. By contrast, only increased but not decreased activity from the norm affected face matching in Experiment 5.

These findings are qualified by another important characteristic of the body language effect that emerged in the current experiments. Whenever body language exerted an influence on person identification, by increasing the number of correct mismatch detections, this was consistently met by a corresponding decrease in match accuracy. Thus, body language did not *improve* the accuracy of person identification here but *biased* this towards the detection of mismatches. Moreover, in contrast to the normal body language conditions, for which identification accuracy correlated with established tests of face matching (i.e., GFMT—Burton et al., 2010; KFMT—Fysh & Bindemann, 2018b), when matching performance was biased by body language, it no longer associated with face-matching ability (Experiment 6).

Several potential explanations exist for this effect. One possibility is that, rather than serving to focus observers’ face-matching resources more strongly on suspicious cases, unusual body language was employed as a simple heuristic that actually undermined the classification of facial information. This finding resonates with the observation that an overemphasis on detecting deception through nonverbal behaviour can result in the adoption of inaccurate stereotypical cues (Vrij et al., 2010). A similar mechanism may underlie the effects observed here, whereby the available facial information to reach identity decisions was surpassed by non-identity-specific body language cues (see Vrij, 2008b).

An alternative explanation could be that higher activity levels in the unusual body language conditions prevented perceivers from extracting the relevant visual information from faces to perform the identification tasks. This would align with research showing that *facial* movement confers no clear benefit in the identification of unfamiliar faces across a range of paradigms (e.g., Bruce et al., 1999; Christie & Bruce, 1998; Darling et al., 2008; Knight & Johnston, 1997) and that matching the identity of moving faces is *worse* than that of static face pairs (Lander et al., 2004). A comparison of different activity levels suggests, however, that this is unlikely to account for the current findings. In Experiment 3, for example, lively and hyper body language produced identical results in comparison with the idle body language condition. This suggest that the difference in activity level between lively and hyper trials did not affect facial identification but only their shared *unusualness* from the body language norm.

### Implications

This study was motivated by the limited research that has been conducted on face matching under the more complex conditions that real-life settings can present and the impact of this on current scientific understanding of person identification at passport control. Most face-matching research in psychology has utilised highly controlled laboratory tasks with images of static face pairs (for a review, see Fysh & Bindemann, 2017a) or field studies that make it challenging to control participants’ behaviour systematically (e.g., Kemp et al., 1997; White et al., 2014). Both approaches therefore carry specific constraints for understanding face matching in more complex settings. The current experiments demonstrate the unique characteristics of VR to complement these approaches, by providing more complex environments than laboratory experiments to examine face perception during social interaction while also retaining experimental control to a greater extent than field studies can afford.

In doing so, this study provides insight into the impact of nonverbal behaviours, such as body language, on facial identification at passport control. Some heuristic techniques to detect identity impostors at passport control have focused on the detection of unusual behaviour, with large-scale programmes in existence to train staff to look for such nonverbal cues (e.g., Screening of Passengers by Observation Techniques; United States Government Accountability Office, 2010). While it is not clear whether this has enhanced aviation security, the current experiments suggest that body language might affect only person identification at passport control when observers are explicitly monitoring variation in such behaviour. And under those circumstances, increases in activity, rather than behaviour that is generally different to the norm, might drive these effects.

This finding converges with work that shows that observers expect increased head and body movements and gaze aversion to indicate deception (Akehurst et al., 1996; Bogaard et al., 2016; Hartwig & Granhag, 2015; Strömwall & Granhag, 2003). There also exists a wider belief that deceptive people are more nervous than truth-tellers, so it is common to interpret increased body language as signs of deception (Vrij, 2008a). These beliefs appear to be stable across professions and laypersons (Akehurst et al., 1996; Bogaard et al., 2016; Vrij et al., 2006), but in reality, no such associations seem to exist (Bogaard et al., 2016; DePaulo et al., 2003; Hartwig & Granhag, 2015; Strömwall & Granhag, 2003; Strömwall et al., 2004; Vrij et al., 2006). For example, in a phenomenon referred to as the *Othello error* (Ekman, 1985/2001), people can also appear nervous when they are expressing the truth, through fear of not being believed or simply from being accused (Vrij et al., 2010). In turn, there is evidence that deceptive behaviour may *actually* be characterised by reduced body movements (Akehurst et al., 1996; Vrij, 2008a; Vrij & Mann, 2001). In this context, it is poignant that reduced body language did not affect identification here. If this finding were to generalise to passport control in real-world settings, then this would imply that, while unusually active body language influences person identification (when observers are actively monitoring for such cues), the body language of *truly* deceptive passengers seeking to avoid detection may not.

In addition, the current experiments also indicate that such body language is utilised in a stereotypical fashion that biases rather than improves the accuracy of person identification. Such biases may be useful in these occupational settings under particular circumstances, for example, where mismatch detection is prioritised irrespective of its cost (such as the false classification of matches) or where mismatch detection is important but (not all) staff are necessarily capable of doing so (e.g., White et al., 2014). In terms of enhancing actual accuracy, however, the current findings support the notion that behaviour detection activities provide an inadequate means to improve aviation security in real-world settings (United States Government Accountability Office, 2013). It remains to be seen, of course, whether similar results would be obtained in this paradigm with passport control staff and other security professionals.

### Limitations

The application of VR to study face matching is a novel and highly exploratory approach that still requires much further development to enhance the person avatars and realism of the VR environment (see Tummon et al., 2019). In the current context, which focuses on the interplay of nonverbal behaviour and face matching, these issues are complicated by the fact that there appear to be no body language cues that uniquely associate with suspicious behaviour (DePaulo et al., 2003; Vrij, 2008a). We circumvented this issue in the current experiments through prior instruction of what constitutes unusual body language (see the Method section in Experiment 3). As a consequence, however, the current effects may simply reflect how unusual body language was implemented. Manipulation of the activity level of the built-in idle modes of avatars provides a very limited proxy for the variety of behaviours that people can exhibit naturally. At the least, however, our manipulation of idle modes meets the operational demands of nonverbal behaviour indicative of deception, of being unusual *somehow* (United States Government Accountability Office, 2010). Another aspect of this body language manipulation is that, because these effects emerged only when participants were given specific instruction to look for these particular nonverbal cues, similar results may be obtained with other behavioural, visual, or auditory cues. This speaks to the generalisability of the current findings but also suggests that these do not necessarily reflect body language per se.

Another potential limitation of the current findings is the small number of trials on which the results are based, with only 6 matches and 6 mismatches of all 100 trials contributing to the main analyses. Unusual nonverbal behaviour is by definition infrequent, necessitating a constraint on the number of trials that were available for this condition. In addition, mismatches also occur much less frequently than identity matches in passport control settings and must therefore share the infrequency characteristic of unusual body language (e.g., Bindemann et al., 2010; Papesh & Goldinger, 2014; Susa et al., 2018). There are several reasons, however, for why the trial count for unusual body language and mismatches cannot explain the current findings. These include the rotation of stimuli around these conditions across participants. The contrasting findings when no instruction (Experiments 1 and 2) or explicit instruction (Experiments 3 to 6) were given to utilise body language further indicate that trial count cannot explain these findings, with the same design employed across all experiments. The difference in findings between Experiments 3 and 5, which employed the same body language conditions but manipulated the activity level of the baseline condition, also emphasises that the current effects are not tied to specific stimuli or the trial count. In turn, the consistent results and the strong influence of body language in Experiments 3 to 6 point to robust effects. Finally, we note that similar approaches to person identification are implemented widely in related fields. In the study of eyewitness memory, for example, experiments routinely comprise of only a single trial to increase ecological validity (e.g., Lindsay et al., 2011; Mansour et al., 2017).

One aspect of our findings that deserves further attention in the context of the small trial numbers relates to the correlations between identification performance in VR with two established laboratory tests of face matching (GFMT and KFMT). When the majority of avatars exhibited idle body language, reliable correlations with these face tests were found, but the effect size was small (mean *r* = .224). This might reflect the limited number of trials from the VR task for this analysis, which can produce only a gross index of participants’ face-matching ability. By comparison, correlations of laboratory-based tests that study face matching with static photographs are in the range of *r* = .4 – .5 (e.g., Fysh & Bindemann, 2018b; Tummon et al., 2019). However, person identification under more realistic conditions contains a range of extrafacial information that is not captured by these laboratory tasks, such as body language, gait, shape, or clothing, which creates additional variability. It is therefore perhaps unsurprising that correlations of the current VR paradigm, which attempts to mimic some of these conditions, are weaker with laboratory-based matching tests. A problem might, in fact, exist in the assumption that laboratory tasks with static face pairs adequately capture real-world person identification tasks (see Bate et al., 2019). The VR approach that is presented here may become an important research tool for bridging this divide, by allowing the study of more complex processes under controlled conditions.

## Figures and Tables

**Table:** 

		Percentage accuracy		Arcsine square root	
Experiment 1	Trial Type	*F* = 43.21	*p* < .001	*F* = 43.23	*p* < .001
ANCOVA	Activity Level	*F* = 0.34	*p* = .72	*F* = 0.34	*p* = .72
	Sensitivity	*F* = 1.45	*p* = .24	*F* = 1.45	*p* = .24
	Trial Type × Activity Level	*F* = 1.01	*p* = .37	*F* = 1.01	*p* = .37
	Trial Type × Sensitivity	*F* = 0.51	*p* = .48	*F* = 0.51	*p* = .48
	Activity Level × Sensitivity	*F* = 0.79	*p* = .46	*F* = 0.79	*p* = .46
	Trial Type × Activity Level × Sensitivity	*F* = 0.97	*p* = .39	*F* = 0.97	*p* = .39
Experiment 2	Trial Type	*F* = 26.53	*p* < .001	*F* = 26.54	*p* < .001
ANCOVA	Activity Level	*F* = 0.02	*p* = .98	*F* = 0.02	*p* = .98
	Sensitivity	*F* = 1.02	*p* = .32	*F* = 1.02	*p* = .32
	Trial Type × Activity Level	*F* = 1.87	*p* = .16	*F* = 1.87	*p* = .16
	Trial Type × Sensitivity	*F* = 0.15	*p* = .70	*F* = 0.15	*p* = .70
	Activity Level × Sensitivity	*F* = 0.92	*p* = .40	*F* = 0.92	*p* = .40
	Trial Type × Activity Level × Sensitivity	*F* = 0.36	*p* = .70	*F* = 0.36	*p* = .70
Experiment 3	Trial Type	*F* = 0.67	*p* = .42	*F* = 0.66	*p* = .42
ANOVA	Activity Level	*F* = 0.08	*p* = .92	*F* = 0.08	*p* = .92
	Trial Type × Activity Level	*F* = 32.83	*p* < .001	*F* = 32.83	*p* < .001
Experiment 4	Trial Type	*F* = 8.44	*p* = .007	*F* = 8.43	*p* = .007
ANOVA	Activity Level	*F* = 2.19	*p* = .12	*F* = 2.18	*p* = .12
	Trial Type × Activity Level	*F* = 48.92	*p* < .001	*F* = 48.93	*p* < .001
Experiment 5	Trial Type	*F* = 2.89	*p* = .10	*F* = 2.88	*p* = .10
ANOVA	Activity Level	*F* = 0.07	*p* = .94	*F* = 0.07	*p* = .94
	Trial Type × Activity Level	*F* = 20.12	*p* < .001	*F* = 20.12	*p* < .001
Experiment 6	Trial Type	*F* = 9.28	*p* = .003	*F* = 7.97	*p* = .006
ANOVA	Activity Level	*F* = 3.24	*p* = .08	*F* = 2.02	*p* = .15
	Trial Type × Activity Level	*F* = 78.58	*p* < .001	*F* = 79.15	*p* < .001

*Note*. ANOVA = analysis of variance; ANCOVA = analysis of covariance.

## Notes

1 Owing to the limited number of critical trials, and to ensure a normal distribution of these percentage accuracy data, an arcsine square root transformation was also applied and the analyses repeated. In this and all subsequent experiments, the same result was obtained, as shown in the Appendix.

2 Specifically, once fitted with the VR headset, participants were read the written instruction: When looking out for mismatches, consider how the people are behaving. While standing in front of the [passport control] desk, the avatars are shifting their stance at different speeds. You should look out for such differences in body language, as these may be useful to detect identity mismatches.
